# Ovarian Response and Fertility after Short-Term Progestagen/eCG Treatments Are Compromised in Nulliparous Sheep during Non-Breeding Season

**DOI:** 10.3390/vetsci9120663

**Published:** 2022-11-28

**Authors:** Zurisaday Santos-Jimenez, Paula Martínez-Ros, Teresa Encinas, Juan Luis Morales-Cruz, Hugo Zuriel Guerrero-Gallegos, Ramiro Gonzalez-Avalos, Antonio Gonzalez-Bulnes, Juan Manuel Guillen-Muñoz

**Affiliations:** 1 Departamento de Farmacologia y Toxicologia, Facultad de Veterinaria, UCM, Ciudad Universitaria s/n, 28040 Madrid, Spain; 2 Departamento de Produccion y Sanidad Animal, Facultad de Veterinaria, Universidad Cardenal Herrera-CEU, CEU Universities, C/Tirant lo Blanc 7, Alfara del Patriarca, 46115 Valencia, Spain; 3 Unidad Laguna, Universidad Autónoma Agraria Antonio Narro, Torreón 25315, Coahuila, Mexico

**Keywords:** anestrous sheep, nulliparous sheep, induction estrus

## Abstract

**Simple Summary:**

The application of treatments based on controlled dose progesterone intravaginal devices (CIDR) plus equine chorionic hormone (eCG) has been favored in recent years for the induction and/or synchronization of estrus activity and ovulation in the breeding and the non-breeding seasons. In anestrous mature ewes, short-term treatments are equally effective as long-term treatments, but information on the effects of such treatments for maiden sheep is scarce.

**Abstract:**

The objective of this investigation was to determine the ovarian response, fertility, and prolificacy of nulliparous sheep when compared to multiparous sheep after a short-term (7 days) CIDR/eCG treatment which was administered during the non-breeding season. All the multiparous sheep, whereas only 54% of the nulliparous ewes, showed signs of estrus. However, 81.8% of the multiparous sheep and 100% of the nulliparous ewes ovulated. Fertility was also low after short-term progesterone treatments during the anestrous season in maiden sheep (30.8 vs. 72.7% in multiparous ewes). Such results indicate significant differences in the response to CIDR/eCG protocols for induction and synchronization of estrus and ovulation between nulliparous and multiparous sheep during the non-breeding season.

## 1. Introduction

The global human population has been significantly growing in recent years and, by the year 2050, will be approximately 9.5 billion people. Such population growth encompasses an increasing demand for food of animal origin. However, keeping in mind the problems related to the scarcity of resources and global warming, the high demand for food needs to be fulfilled with fewer alterations to the environment and to taking care of animal welfare parameters [1,2,3,4].

In such a scenario, small ruminants are a major economical and sustainable resource for rural people living in developing regions and transition countries [5] and in adverse climatic conditions or in harsh and sub-fertile areas, where the breeding of other animals is highly inefficient [6].

In such conditions, health, nutrition, and reproductive efficiency are critical issues for sheep breeding. Sheep are a short-day, seasonally polyestrous species, [3,7,8], with periods of anestrus, which are modulated by exogenous factors (environmental temperature, nutritional status, and social interactions) [9,10,11].

Such patterns affect the availability of sheep products during the year and make necessary the induction of reproductive activity during the seasonal anestrus. [12]. The reproductive management during non-reproductive seasons is mainly based on the use of exogenous progesterone during 12–14 days, combined with equine chorionic gonadotrophin (eCG) for the stimulation of terminal follicular development [13,14,15] and the avoidance of influence of anestrus [16,17].

Currently, ultrasonographic evidence on follicle growth patterns along with health and welfare issues have resulted in the shortening of progesterone-based protocols (5–7) regarding days of treatment [18,19,20]. Short-term protocols are now frequently used for artificial insemination of sheep under field conditions, although they are still far less popular among producers than classical long-term treatments [21]. The main causes for the reluctance of breeders to use short-term protocols are the need for a PGF2a injection, which causes additional costs (although costs imposed by longer treatment periods and reproductive cycles are not considered) and their own routine using yearly long-term treatments. However, short-term protocols are as effective as long-term protocols for inducing fertile estrous and ovulation in both breeding and non-breeding seasons [18,19,20].

These data were, however, obtained in multiparous sheep and there is, to the best of our knowledge, a lack of data for nulliparous sheep. The success of protocols for cycle management during the non-breeding season may be compromised in maiden females [22]. First, there are problems associated with the effects of the photoperiod at the hypothalamic level, which is reflected in the absence of endogenous LH during anestrus [23], which may be also compromised by the maturational changes associated with puberty [17,24,25,26,27]. Second, initial ovulations at the onset of puberty are generally not accompanied by estrus [16], nor occur after very short-in-duration estrus [28]. Progesterone inhibits the pulsatile secretion of the gonadotrophin releasing hormone (GnRH), and thus LH, in an opposite effect to the positive feedback of estradiol (E2) on the secretion of GnRH and LH [29]. Hence, during the luteal phase of the cycle, when progesterone concentrations are high, the frequency of the GnRH/LH pulses is low. The decrease in progesterone concentration after luteolysis allows the GnRH/LH pulse frequency to increase when stimulated by increased estrogen concentrations [30]. We hypothesize that the use of progesterone-based devices for a short time should overcome this lack of endogenous progesterone, and in a similar way as described for long-term treatments [31,32]. However, there is no previous knowledge on the yields obtained after using such short-term treatments.

Therefore, the objective of this present study was to determine the ovarian response, fertility, and prolificacy of nulliparous sheep when compared to multiparous sheep, after the administration of a short-term CIDR treatment during the non-breeding season.

## 2. Materials and Methods

### 2.1. Animals, Ethical Issues, and Experimental Design

All the experimental procedures in the current study were performed according to national and international standards [33,34], respectively, for the ethical care and protection of animals used in research.

The trial was carried out during the non-breeding season (March) under natural photoperiod conditions in an intensive production system in a commercial farm (Coahuila, Mexico; latitude 25°37′ N and longitude 103°23′ W). The rainfall in the month of the experiment was 0.2 mm with maximum average temperatures of 28.4 °C and minimum average temperatures of 11.2 °C. Twenty-four clinically healthy Dorper ewes were involved in the study, being either nulliparous (*n* = 13; around one year-old with no previous births, an average body-weight of 35.7 ± 0.8 kg, and a body condition score of 3.1 ± 0.20; mean ± S.E.M) or multiparous, with a range of 2 to 4 births, and having an interval to the previous lambing that was greater than three months (*n* = 11; average body weight of 44.0 ± 0.15 kg and body condition score of 2.5 ± 0.10; on a scale of (0 = very thin, and 5 = very fat) [35]. The sheep were housed in shaded pens. The males were separated from the females in individual pens 500 m away from the females. All animals had free access to water and alfalfa (comprising 17% crude protein and 1.95 Mcal metabolizable energy).

Seasonal anestrus was confirmed in all sheep by ultrasonographic ovarian scanning with a B-mode 7.5 MHZ transrectal linear transducer (Eco 5, Chison Co., Wusi, China). The technician introduced the transducer rectally, previously lubricated with a water-based lubricant, at an angle of 45°. Assessment of the ovaries and characterization of the ovarian structures was performed as described by González–Bulnes et al. [36] for ruling out the presence of corpora lutea. Two scans were performed with an interval of 7 days, with the second before the insertion of the device to ensure that the sheep were anovulatory, according to previous studies in the region [37]. The treatment included the insertion of one intravaginal CIDR device containing 0.3 g of progesterone (CIDR^®^ Ovis, Zoetis, Mexico City, Mexico) for seven days. On CIDR withdrawal, all the females were i.m. treated with 5 mg of prostaglandin F2α (Lutalyse, Zoetis, Mexico City, Mexico) and 300 I.U. of eCG (GonActive^®^ eCG, Virbac, Zapopan, México).

The variables evaluated during the induced follicular phase and the subsequent luteal phase were the percentage of animals displaying estrus and the timing of estrous behavior, development of ovulatory follicles, and the timing of their ovulations, number of induced corpora lutea and pregnancy rate, and number of embryos in response to the treatment.

### 2.2. Occurrence and Timing of Estrous Behavior

Signs of estrus behavior were determined twice daily (every 12 h) for five days after CIDR removal. A trained ram was introduced to the group of females for around 15 min. Each female that was identified as being in estrus was taken out of the pen and led to be mated by another male on a one ram/one ewe basis. The intervals from treatment to the onset and ending of the estrus, and therefore the duration of estrus, were defined by the time of the first accepted mating to the first refusal for mating.

### 2.3. Occurrence and Timing of Ovulation

The females that showed signs of estrus underwent assessment of the number and development of follicles by transrectal ultrasonography (Eco 5, Chison Co., Wusi, China). Once the ovary was located, the disappearance of a large ovulatory follicle (circular anechoic structures representative of follicles with ≥4 mm in size) was used as marker of occurrence of ovulation [38]. Ovarian observations were carried out every 12 h; from 36 to 84 h after CIDR removal.

### 2.4. Ovulation Rate

On day 10, after device removal, presence and number of corpora lutea compatible with ovulation after the treatment were recorded by transrectal ultrasonography in all females [36].

### 2.5. Fertility and Prolificacy

The occurrence of pregnancy and the number of embryos were evaluated by transrectal ultrasound on day 35 after CIDR removal. In this observation, the anechoic structures in the uterus, compatible with embryo sacs were counted, so females with these characteristics were considered pregnant.

### 2.6. Statistical Analysis

Data were firstly analyzed by a Shapiro test to determine the normality, and afterwards, the homogeneity of variances was analyzed by a Bartlett test. The data that did not follow a normal distribution were transformed using the cosine function. The statistical model to analyze the variables of duration of estrus (h), time of onset of estrus after CIDR removal (h), ovulation time after CIDR removal (h), ovulation rate (*n*), diameter (mm), and number of follicles (*n*) were analyzed by a comparison of means through a Student’s *t*-test. Occurrence of estrous behavior (%), distribution of females showing estrus (%), occurrence at ovulation (%), fertility rate with respect to treated females (%), and fertility rate with respect to females that ovulated (%) were analyzed using a chi-square test. All results in the main text and tables are expressed as mean ± S.E.M. and statistical significance was accepted from *p* < 0.05. All the procedures were executed with the R program Version 4.0.5 (The R Foundation for Statistical Computing, Boston, MA, USA).

## 3. Results

**Figure 1:**
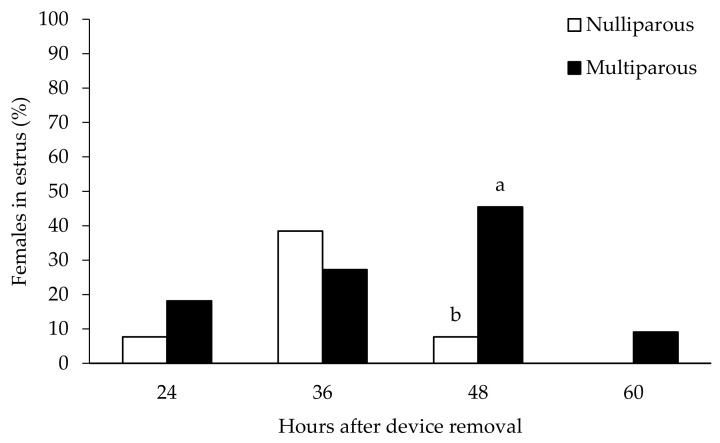
Distribution (%) of sheep showing appearance of estrous behavior over time after CIDR removal. Different letters indicate statistical differences between groups (a ≠ b: *p* < 0.05).

There were significant differences in the response of nulliparous and multiparous females to the treatment for estrus synchronization (Table 1). In brief, all the multiparous ewes showed estrous activity whereas only around half of the maiden ewes responded to the treatment displayed heat signs. There were no significant differences in the timing of onset and duration of such estrus signs between groups, but the distribution of estrus onset was narrower in maiden ewes (24 to 36 h after CIDR removal) than in adults (24 to 60 h after CIDR removal), as depicted in Figure 1.

**Figure 2:**
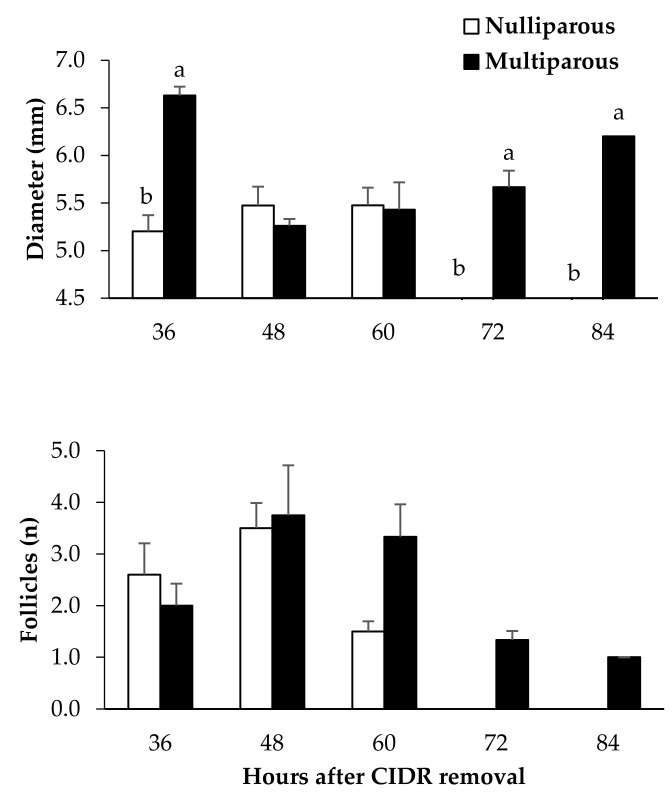
Mean (±) follicle number and diameter in ewes that showed estrous activity after CIDR removal. Different letters indicate statistical differences between groups (a ≠ b: *p* < 0.05).

These features also correspond to differences in the patterns of preovulatory follicle development between nulliparous and multiparous sheep (Figure 2). All the maiden ewes showed a disappearance of preovulatory follicles after 60 h from CIDR removal. The mean diameter of the preovulatory follicle at the time of ovulation showed no statistical differences between multiparous and nulliparous groups (5.33 ± 0.20 and 5.46 ± 0.18, respectively; *p* > 0.059). Hence, evidence of ovulation was found in all the nulliparous ewes, and without significant differences with multiparous sheep in timing of such ovulation and in the number of corpora lutea.

Our data show that 46.1 (5/13) of nulliparous ewes had a silent estrus, which was confirmed when assessing ovulation, because all the nulliparous ewes showed a corpus luteum (Table 2). Corpora luteum diameter was greater by 0.35 mm between multiparous vs. nulliparous ewes (*p* < 0.05). Finally, the assessment of pregnancies evidenced a significant drop in the fertility and number of embryos in the nulliparous sheep.

## 4. Discussion

The results of our current study indicate significant differences in the response of nulliparous females to progesterone-based treatments for the synchronization of estrus and ovulation when compared to multiparous sheep. These results are opposite to data published by Ungerfeld and Rubianes [39], who reported a similar response in nulliparous and multiparous sheep with both short- and long-term progestagen treatments during seasonal anestrus.

In our study, performed during the anestrous season, multiparous ewes showed a good ovarian response to the treatment, with all of them displaying estrus after treatment removal, around 82% of them displaying corpora lutea indicative of a good ovulatory process, and with around 75% of them becoming pregnant (around 90% of the multiparous sheep ovulated in response to the treatment). These results are similar to available data in other studies with the same or other breeds [31,32,40,41] and support a good response of Dorper sheep to short-term, progesterone-based protocols when including eCG during the anestrous season [37]. Conversely, other studies have shown that the pregnancy rate may be improved by a longer progesterone imprinting by using long-term protocols (83.3% after 14 days of treatment vs. 60% after 9 days vs. 47.8% after 5 days) [23].

Conversely, a high percentage of the nulliparous sheep failed to develop estrous behavior after progesterone removal (around 45% of them), despite all the treated maiden ewes ovulating afterwards. Hence, the treatment that included progesterone and eCG was successful for inducing estrous behavior and ovulation in adult ewes during the anestrous season, but the response of maiden ewes was affected by occurrence of silent ovulations. Silent ovulations are usual in sheep, and even more in nulliparous sheep at the onset of estrous activity after seasonal anestrus [9,42,43], as it happened in our current study. This event has also been reported even after long-term treatments [44] and is hypothesized to be related to deficiencies in the terminal follicular growth [45]. In the present study, a significantly small size of the preovulatory follicles in maiden sheep at the start of the study supports such a hypothesis, which has been related to a lack of progesterone signaling during the transition of anestrus to ovulatory cyclic activity [46]. We observed that nulliparous ewes showed a smaller corpus luteum size at the time of ovarian assessment at day 10. It is well studied that an inadequate follicular development leads to the formation of a subnormal corpus luteum, which results in low levels of progesterone secretion [47].

Obviously, such silent estruses affected fertility rate and only 30% of the sheep in the group were found to be pregnant. Moreover, we must remark that (a) around half of the maiden sheep displaying estrus signs and being mated failed to become pregnant, and (b) that the relationship between the number of corpora lutea and the number of embryos was low by itself and clearly lower than in multiparous sheep. Such features may be also related to the lack of progesterone signaling, which characterizes the first cycles of maiden ewes. In this sense, the low fertility of young ewes after progesterone treatments is well known, even during the reproductive season [48]. This event has been related to a dramatic decrease in the developmental competence of the oocytes for developing a viable embryo in the maiden ewes when compared to mature sheep [49].

It is also well known that as sheep mature and the effect of the photoperiod decreases (from longer to shorter days), the frequency of GnRH and LH pulses increases, which increases the synthesis and secretion of estradiol by the ovaries [27], which is, in turn, necessary for the manifestation of estrus. Such a photoperiodic pattern affects spontaneous reproductive activity during the year by modifying the functionality of the hypothalamus–hypophysis–ovarian axis by a significant decrease in luteinizing hormone (LH) secretion which, in turn, impedes cyclic ovulatory activity [24,50] and female fertility by affecting the functionality of the hypothalamus–hypophysis–ovarian axis, cyclic ovulatory activity, the quality of preovulatory follicles/oocytes/embryos, and/or subsequent embryo/fetal viability [51]. Although nulliparous ewes were exposed to a pretreatment of progesterone plus eCG, the effects of the photoperiod decreased the reproductive response of the young females in our study.

## 5. Conclusions

Our results indicate a disturbed ovarian response and a low fertility of maiden sheep after a short-term progesterone treatment during the anestrous season. Such results preclude the use of these protocols for induction and synchronization of estrus and ovulation in nulliparous sheep during the non-breeding season under the conditions of our experiment.

## Figures and Tables

**Table 1:** Percentage and timing (± S.E.M.) of occurrence of estrus in multiparous and nulliparous ewes after a short-term treatment with a controlled internal drug release (CIDR) and equine chorionic gonadotrophin (eCG) during seasonal anestrus.

	Multiparous	Nulliparous
Occurrence of estrous behavior (%)	100 (11/11) ^a^	53.9 (7/13) ^b^
Estrus duration (h)	29.5 ± 4.08	24.0 ± 2.52
Time of onset of estrus after CIDR removal (h)	41.5 ± 3.4	36.0 ± 1.9

Different letters indicate significant differences between groups (a ≠ b: *p* < 0.05).

**Table 2:** Percentage and timing (±S.E.M.) of occurrence of ovulation, number of corpora lutea and fertility, and number of embryos in multiparous and nulliparous ewes after a short-term treatment with a controlled internal drug release (CIDR) and equine chorionic gonadotrophin (eCG) during seasonal anestrus.

Variables Evaluated	Multiparous	Nulliparous
Occurrence of ovulation (%)	81.8 (9/11)	100 (13/13)
Time of ovulation after CIDR removal (h)	70.9 ± 3.0	63.4 ± 1.6
Ovulation rate (number of corpora lutea)	1.7 ± 0.3	2.2 ± 0.2
Diameter CL (mm)	12.31 ± 0.5 ^a^	11.96 ± 0.28 ^b^
Fertility rate with regard to ewes treated (%)	72.7 (8/11) ^a^	30.77 (4/13) ^b^
Fertility rate with regard to ewes estrus (%)	72.7 (8/11)	57.1 (4/7)
Fertility rate with regard to ewes ovulating (%)	88.9 (8/9) ^a^	30.77 (4/13) ^b^
Numbers of embryos	1.6 ± 0.2	1.1 ± 0.1

Different letters indicate significant differences between groups (a ≠ b: *p* < 0.05).

## Data Availability

Data are contained within the article.
